# Taking Healthy Steps: rationale, design and baseline characteristics of a randomized trial of a pedometer-based internet-mediated walking program in veterans with chronic obstructive pulmonary disease

**DOI:** 10.1186/1471-2466-14-12

**Published:** 2014-02-03

**Authors:** Carlos H Martinez, Marilyn L Moy, Huong Q Nguyen, Miriam Cohen, Reema Kadri, Pia Roman, Robert G Holleman, Hyungjin Myra Kim, David E Goodrich, Nicholas D Giardino, Caroline R Richardson

**Affiliations:** 1 Pulmonary & Critical Care Division, University of Michigan Health System, 3916 Taubman Center, Box 0360, 1500 E. Medical Center Drive, 48104 Ann Arbor, MI, USA; 2 Pulmonary and Critical Care Medicine Section, VA Boston Healthcare System, 1400 VFW Parkway, 02132 West Roxbury, MA, USA; 3 Division of Pulmonary and Critical Care Medicine, Department of Medicine, Brigham and Women’s Hospital, Boston, USA; 4 Department of Research and Evaluation, Kaiser Permanente Southern California, 100 S. Los Robles Ave, 91102 Pasadena, CA, USA; 5 Patient Services, VA New York Harbor 800 Poly Place, 11209 Brooklyn, NY, USA; 6 Center for Clinical Management Research, VA Ann Arbor Healthcare System, 2215 Fuller Rd, 48105 Ann Arbor, MI, USA; 7 School of Public Health, University of Michigan, 915 E. Washington, 48109-2399 Ann Arbor, MI, USA; 8 Department of Psychiatry, University of Michigan, 2215 Fuller Rd, 48109-2399 Ann Arbor, MI, USA; 9 Department of Family Medicine, University of Michigan, 1018 Fuller St, 48104-1213 Ann Arbor, MI, USA

**Keywords:** COPD, Chronic bronchitis, Emphysema, Quality of life, Exercise, Physical activity, Internet, Pedometer, Walking, Veterans

## Abstract

**Background:**

Low levels of physical activity are common in patients with chronic obstructive pulmonary disease (COPD), and a sedentary lifestyle is associated with poor outcomes including increased mortality, frequent hospitalizations, and poor health-related quality of life. Internet-mediated physical activity interventions may increase physical activity and improve health outcomes in persons with COPD.

**Methods/Design:**

This manuscript describes the design and rationale of a randomized controlled trial that tests the effectiveness of Taking Healthy Steps, an Internet-mediated walking program for Veterans with COPD. Taking Healthy Steps includes an uploading pedometer, a website, and an online community. Eligible and consented patients wear a pedometer to obtain one week of baseline data and then are randomized on a 2:1 ratio to Taking Healthy Steps or to a wait list control. The intervention arm receives iterative step-count feedback; individualized step-count goals, motivational and informational messages, and access to an online community. Wait list controls are notified that they are enrolled, but that their intervention will start in one year; however, they keep the pedometer and have access to a static webpage.

**Discussion:**

Participants include 239 Veterans (mean age 66.7 years, 93.7% male) with 155 randomized to Taking Healthy Steps and 84 to the wait list control arm; rural-living (45.2%); ever-smokers (93.3%); and current smokers (25.1%). Baseline mean St. George’s Respiratory Questionnaire Total Score was 46.0; 30.5% reported severe dyspnea; and the average number of comorbid conditions was 4.9. Mean baseline daily step counts was 3497 (+/- 2220).

Veterans with COPD can be recruited to participate in an online walking program. We successfully recruited a cohort of older Veterans with a significant level of disability including Veterans who live in rural areas using a remote national recruitment strategy.

**Trial registration:**

Clinical Trials.gov NCT01102777

## Background

### Burden and management of COPD

Chronic Obstructive Pulmonary Disease (COPD) is a significant problem worldwide, affecting 9-10% of the population 40 years of age or older [1,2]. In the U.S., COPD is currently the third cause of mortality [3] and affects 5.1% of the population. Among Veterans the prevalence is 8.2% [4] with those afflicted by the disease accumulating more emergency room and outpatient visits to physicians when compared to persons without COPD. COPD is characterized by acute exacerbations (AECOPD), which are responsible for 70% of the total cost generated by the disease. AECOPD accelerate the rate of decline of lung function [5] and negatively impact health-related quality of life (HRQL); nearly 50% of the patients will continue to need help with their activities of daily living six months after hospital discharge for an AECOPD [6]. AECOPD are part of a downward spiral in functionality and HRQL and are also the main risk factor for new or recurrent exacerbations [7].

The principles of COPD management include control of symptoms, prevention of AECOPD, and improving HRQL. Strategies include smoking cessation, pharmacological therapy with short- and long-acting bronchodilators and anti-inflammatory inhaled medications, oxygen supplementation, and early recognition of symptoms. Almost all available therapeutic options have been proven to reduce symptoms, decrease the frequency of exacerbations, and improve HRQL [8-11], but none of the available pharmacologic interventions modifies the progressive loss of pulmonary function [12]. The recognition of a high frequency of cardiovascular disease as cause of death in COPD patients [13] and the role of comorbid conditions as risk factors for recurrent exacerbations has strengthened the interest in the management of other coexistent conditions as an alternative to improve the prognosis of patients with COPD [14], or to develop COPD self-management programs [15]. Engagement in physical activity is a cornerstone of self-management programs to improve health status.

### Exercise and physical activity in COPD

Persons with COPD have significant reductions in physical activity compared to controls [16,17]. Comorbidities of cardiovascular disease, musculoskeletal disease, osteoporosis [18], loss of skeletal muscle mass [19], muscle weakness, and weight loss [20] contribute to loss of functional capacity. Epidemiological and cross-sectional data have shown that physical activity is related to outcomes in COPD. Those who have higher levels of physical activity have a lower risk of hospital readmissions, acute exacerbations, and COPD-related hospitalizations [21]. Physical activity levels are the strongest predictor of mortality, independent of lung function [22].

This association between physical activity and COPD outcomes may be mediated through improved cardiorespiratory fitness or through reduced systemic inflammation [23]. Despite the benefits of physical activity, few interventions exist to promote physical activity in persons with COPD. Pulmonary rehabilitation has been clearly shown to improve exercise capacity, reduce dyspnea, and improve HRQL in persons with COPD [24]. Pulmonary rehabilitation includes exercise training for deconditioning, disease management education, management of dyspnea, appropriate use of medication, and identification of depression and other barriers to exercise. Pulmonary rehabilitation is a potent intervention. The number-needed-to-treat (NNT) with pulmonary rehabilitation to prevent one hospital admission is 4 (95% CI 3–8) [25].

### Limitations to exercise among COPD patients

Unfortunately, pulmonary rehabilitation programs are typically hospital based and are not accessible to everyone who needs them. In addition, the benefits gained after a typical 12-week supervised program plateau and begin to diminish after 9–12 months [26]. In a national survey of COPD patients more than 25% were not aware of the existence of rehabilitation programs, and an additional 13% reported that pulmonary rehabilitation programs were not available [27]. Criteria for participation in rehabilitation programs are usually determined by third party payers, and insurance could be a factor in receiving the intervention. Individual barriers identified in some studies include the need to make changes in the daily routine, competing needs and duties, and transportation problems [28]. Social isolation and living in remote areas is common in COPD patients, with up to 28% of Veterans with COPD residing in rural and isolated areas [29]. Living in rural or isolated areas is associated with a lower probability of receiving home health services and less frequent visits to physicians [30]. Finally, a systematic review identified lack of support and program-specific barriers as factors limiting participation in rehabilitation, while belief in personal benefits and setting goals are enablers for participation [31]. As a result, less than 13% of the potential candidates who might benefit from rehabilitation are referred by their health care provider [32]. Development of individual, home-based physical activity programs may address some of the barriers and may expand the reach to those living far from facilities that offer formal pulmonary rehabilitation programs. The Internet and other new forms of information and communication technology could provide a way to reach this population. A few studies have demonstrated the feasibility, acceptability, and efficacy of Internet-mediated smoking cessation and dyspnea management interventions in COPD [33,34]. Additionally, we have demonstrated the accuracy of the Omron HJ-720-ITC in a pilot study of 24 patients with COPD. At walking speed the pedometer was able to capture more than 80% of manual step counts, and the patients were able to upload the information [35]. In another study with 90-day follow-up, 27 older Veterans (mean age 72, s.d. 8 years) demonstrated the ability to regularly upload pedometer step counts to their home computer, and to be part of a feedback Internet-mediated program. They were also satisfied with the intervention and would recommend a similar program to potential participants [36].

Based on the previously reviewed evidence of the benefits of exercise as part of rehabilitation programs and recognizing the multiple barriers to participation in exercise programs faced by older patients, we designed the current pedometer-based, Internet-mediated intervention to promote walking in this population. The long-term objectives are to develop, test, and disseminate effective, low-cost interventions that improve HRQL for Veterans, particularly rural-living Veterans managing complex chronic conditions, which could be extrapolated to other similar populations.

## Methods

### Study design

Taking Healthy Steps is a randomized controlled clinical trial for Veterans with COPD across the U.S. The coordinating center is located at the VA Center for Clinical Management Research in Ann Arbor, Michigan.

### Conceptual framework

**Figure 1:**
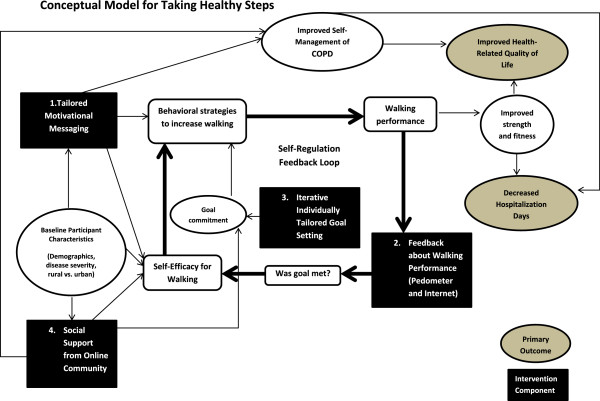
Conceptual Model for Taking Healthy Steps.

The Taking Healthy Steps intervention is based primarily on self-regulation theory. The Theory of Self-Regulation emphasizes an iterative, rational process of behavior change in which an individual working towards a behavioral goal learns from successes and failures and uses this knowledge to develop effective behavioral strategies to achieve his or her goal [37,38] (Figure 1). Accurate self-monitoring, goal setting, and feedback are critical components of the cycle of self-regulation. The Taking Healthy Steps intervention targets the cycle of self-regulation with four components: 1) step-count feedback from a pedometer and personal website, 2) automated, gradually incrementing goals, 3) tailored motivational messages, and 4) an online community to enhance social support. Participants upload detailed time-stamped step-count data. Tailored algorithms based on the data provide dynamic individualized incremental walking goals and feedback about success at meeting these goals. Goal setting is based on Lock and Latham’s demonstration that high, hard goals improve performance as long as the goals are not too high [39]. Goal increment is based on a series of pilot studies in individuals with a variety of chronic illness [40]. Online communities allow users to interact with each other by posting messages for others to read and by reading messages posted by others. These communities are useful for individuals who report low baseline social support, which is a predictor of poor adherence to physical activity interventions.

### Study aims

The specific aims of the current clinical trial are:

1. To test the effectiveness of an automated, internet-mediated walking program on HRQL at four months and at one year

2. To estimate the effect of the Taking Healthy Steps program on all cause days of hospitalization over one year in Veterans with COPD

3. To compare intervention reach, participation, and satisfaction between rural and urban Veterans among those randomized to the intervention arm

### Identification of target population

**Figure 2:**
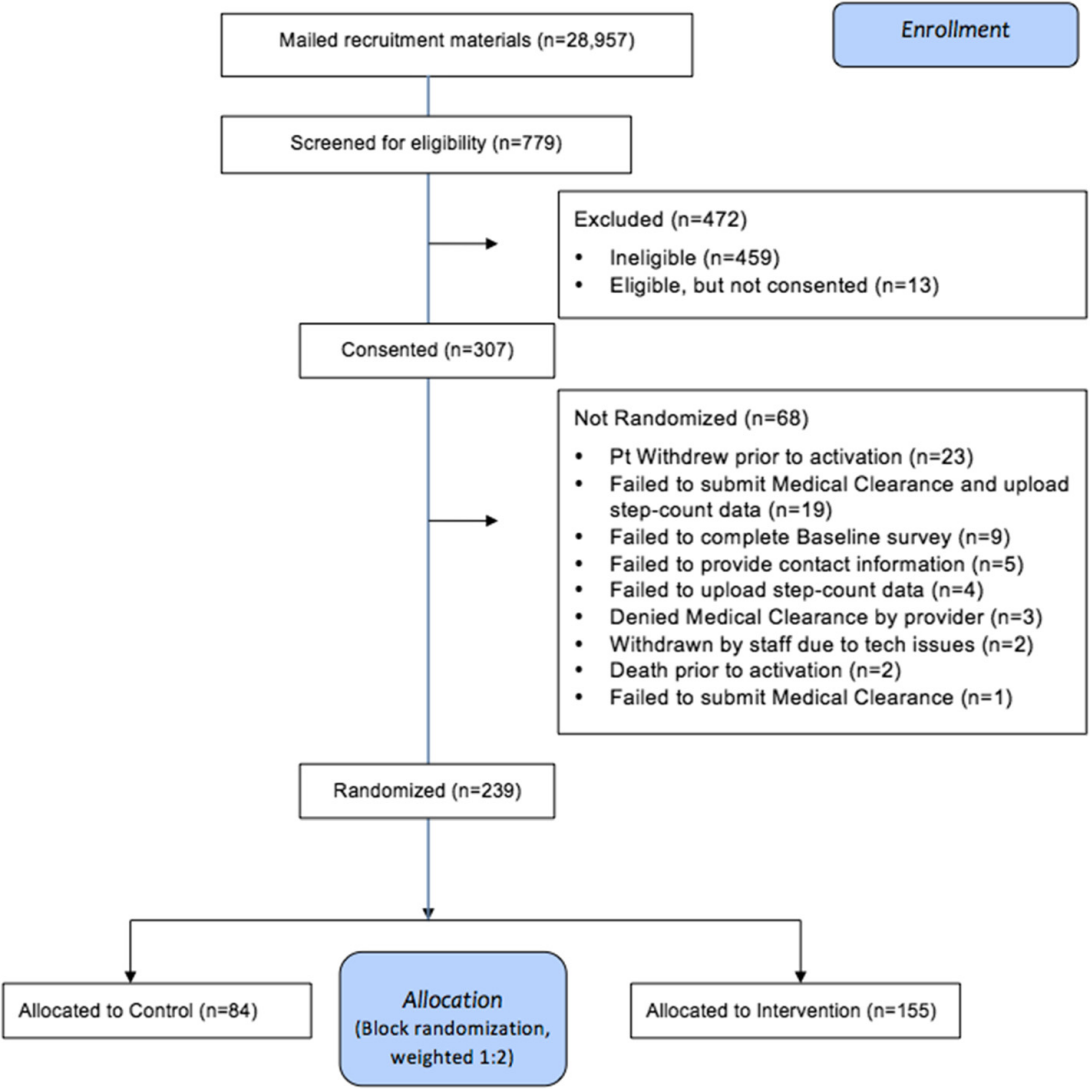
CONSORT diagram of the enrollment and allocation in the Taking Healthy Steps clinical trial.

The flow of patients through the study is described in Figure 2. Potential participants are identified from VA national databases of patients who received care within the year prior to enrollment from a primary care provider, cardiologist, or pulmonologist within the VA health care system. The diagnosis of COPD is based on data extracted from ICD9 codes of 491.x Chronic Bronchitis, 492.x Emphysema and 496.x Chronic Airway Obstruction NEC indicating the likely presence of COPD. The identification of COPD patients using a similar algorithm has been associated with appropriate diagnostic accuracy (area under the ROC curve 0.75) [41]. Veterans less than 40 years old (to minimize misclassification with asthma) and individuals with diagnosis codes for quadriplegia and paraplegia, wheelchair dependence, dementia, or pregnancy-related diagnoses or procedures within the previous year were not eligible. We also excluded Veterans from VISN 01 facilities, where another COPD study using the Taking Healthy Steps platform was actively recruiting participants. After identifying potential participants, their zip codes were matched to Rural Urban Commuting Codes (RUCA), a validated algorithm developed by the U.S. Department of Agriculture – Economic Research Service to classify U.S. census tracts using measures of population density, urbanization, and daily commuting [42], which identified potential participants as living in urban or rural areas.

Based on reported response rates of 2-5% in similar surveys of COPD patients, a random subset of 30,000 Veterans (half urban and half rural) was selected from the pool to receive mailed invitations to participate in the program. The invitation letter includes a brief description of the study and key eligibility criteria, as well as a website address to visit for more information, a six-digit numeric ID code to enter into the online form that allows them to proceed with further eligibility screening, and a pamphlet about research within the VA system. This remote recruitment strategy was intentionally designed to expand the reach to rural Veterans. Letter recipients who visit the website read an expanded description of the study. If interested, they enter their email address into a web form and complete an online screening questionnaire that assesses their demographics, health status, physical activity level, and computer access.

### Eligibility

Eligibility requirements include: Veteran; age ≥ 40 years old; diagnosis of COPD, emphysema, or chronic bronchitis based on administrative ICD-9 codes or self-report; able to walk a minimum of one block; sedentary (defined by < 150 minutes of self-reported physical activity per week); have a doctor or primary care provider who can give medical clearance; competent to give informed consent, and not having a legal guardian; regular email user (checking weekly); have access to a computer with an Internet connection, a USB port, and Windows XP, Vista, 7, or 8; not involved in another pedometer-based walking program.

Upon submission of the eligibility survey responses, an algorithm determines eligibility in real time. The website then directs eligible individuals to an online consent form with a click-through agreement and the opportunity to have study staff contact them to answer questions. Eligible participants present a medical clearance form to their health care provider to receive confirmation that the walking program is safe for them, and the signed form is faxed back to the coordinating center. A similar procedure of medical clearance has been previously tested in other studies by our group [43].

### Baseline measures and randomization

After completing the online screening and informed consent, participants complete online surveys about the primary outcome of HRQL using the St. George’s Respiratory Questionnaire and the general health status question from the SF-36. The surveys also assess demographics; health history including comorbidities and COPD hospitalizations; motivations and barriers for walking; knowledge and attitudes about COPD, current cigarette smoking, current supplemental oxygen use, the Modified Medical Research Council (MMRC) dyspnea score, depression, social support; and comfort with computers. Once the baseline survey is complete, study staff mail participants an Omron pedometer (HJ-720 ITC) with a USB upload cable and instructions about uploading pedometer data and pedometer care and use. This pedometer has been previously validated in a similar population [44]. Participants are asked to wear their pedometers throughout the day for seven days to assess their baseline step counts. Pedometers have a sticker over the display during the baseline assessment period, and no step-count goals or feedback are provided during this stage. At the end of the seven days the participant uploads their pedometer data using the USB cable and their computer. Immediately after completion of baseline pedometer data collection and receipt of the medical clearance, the participants are automatically randomized to one of two arms: 1) Internet-mediated, pedometer-enhanced program (Taking Healthy Steps) or 2) Wait list control. Randomization is stratified by breathlessness (defined by MMRC) and urban vs. rural residence.

### Pedometer-based, internet-mediated intervention (Taking Healthy Steps)

**Figure 3:**
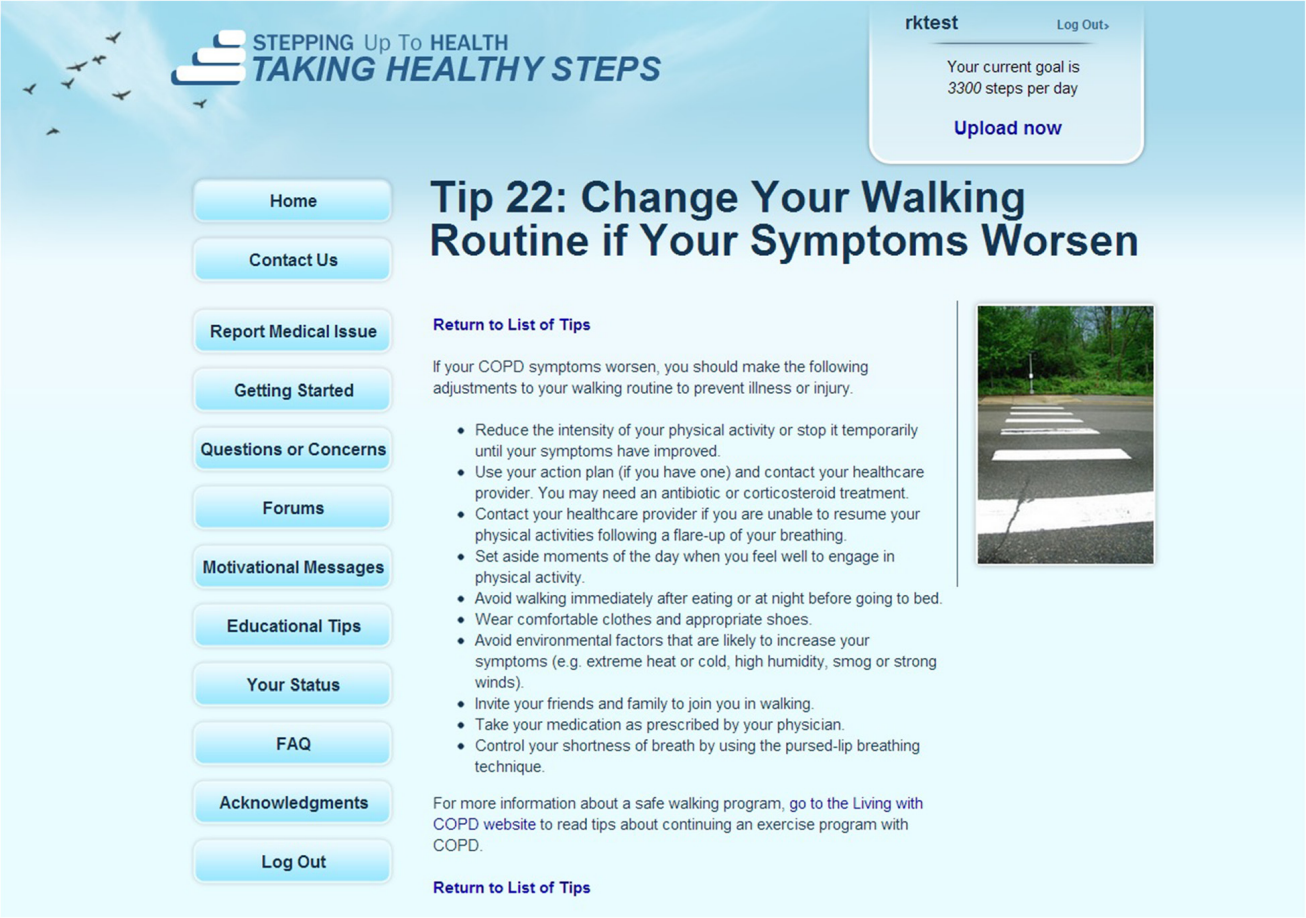
Example of the informational messages provided for participants in Taking Healthy Steps.

After being randomized to Taking Healthy Steps, participants receive an automated email message informing them of their first step-count goal and instructing them to remove the sticker from their pedometer. They also gain full access to their personal home page with individualized step-count goals, step-count feedback, graphs, and motivational messages. Participants receive two kinds of *tailored messages* on the website: a weekly motivational and an every-other-day informational message. The weekly *motivational* messages are based on individual responses to the baseline survey and address specific characteristics such as oxygen use and cigarette smoking, motivations, and barriers reported by the individual participant, highlighting benefits of exercise while addressing perceived personal barriers and strategies to overcome those barriers. A new *informational* message appears on the study home page every other day; some of these messages contain disease-specific content related to COPD while others are generic information about walking and health. Disease-specific content includes education about the potential benefits of walking for persons with COPD and overcoming COPD-specific barriers to walking (fear of becoming short of breath, fear of running out of oxygen in a public place, embarrassment with use of oxygen tank in public places). In addition, information is provided on specific problems (use inhaler before starting out on a walk), anticipated problems (walk an unknown course with a spouse first, walking on different surfaces and during different seasons), and building confidence in participating in a regular walking program (walk a specific route that you know you can complete without becoming short of breath). Where possible, content is evidence-based and modeled on content used in previously published COPD self-management and pulmonary rehabilitation studies. An example of the messages is displayed in Figure 3. Both the motivational and informational messages address barriers to walking that we have identified as being significant to COPD patients in a similar trial [45].

Participants are asked to wear the pedometer every day, from waking to sleeping, during the initial 16-week period of intervention, and are encouraged to continue wearing and uploading the data throughout the eight-month maintenance phase.

*Feedback* is provided in three ways: 1) participants can read their step-count data from the pedometer display throughout the day; after uploading step-count data to the website, they can select from a variety of detailed graphs displaying different views of their step counts, including weekly and daily views with daily or hourly step-count totals; 2) participants can look back at their graphs and goals from previous weeks; and 3) the website displays a textual message about their step-count average for the week.

**Figure 4:**
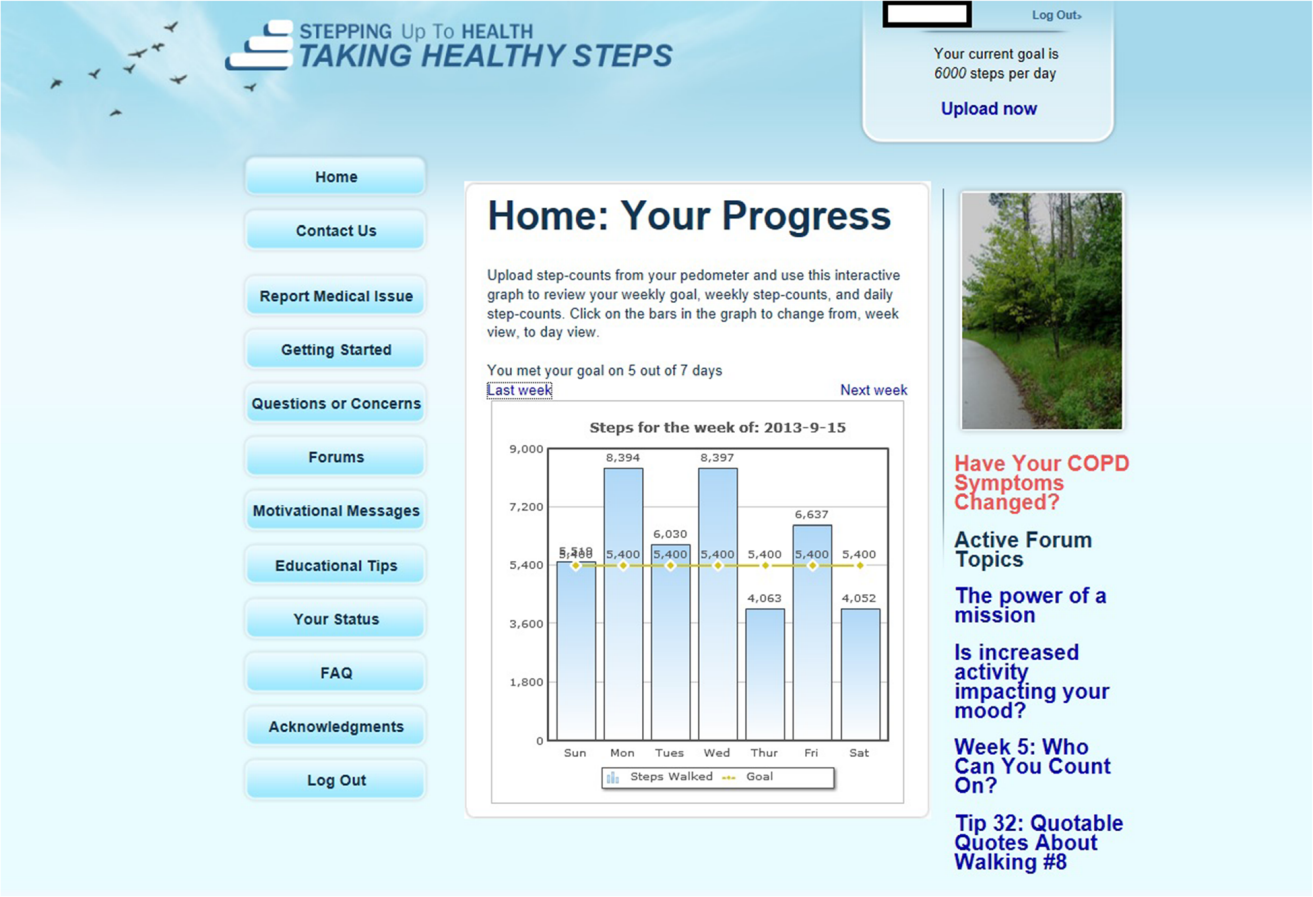
Example of the step-counts screen for participants in Taking Healthy Steps.

*Goal-setting* is provided each week for the entire one-year intervention period, with participants receiving a new individually tailored and automatically calculated daily step-count goal. The algorithm for calculating step-count goals was developed based on a series of usability studies, pilot studies, and RCTs in participants with type 2 diabetes, coronary artery disease, and obesity [40,46]. This algorithm has been validated for use in similar populations [43]. The algorithm involves adding a fixed increment to the average step counts of uploaded pedometer data for the participant over the previous seven days. Because COPD patients tend to be less physically active and may find it more challenging to increase their step counts, the goal is never higher than a 600-step increment, rather than the 800- or 1200-step increments reported for other populations. Goals are rounded off to the nearest 100 steps, and the maximum possible goal is 10,000 steps per day. Each Sunday, the goal calculation algorithm is run and sends participants automated emails with their personalized daily step-count goal for the week, which appears in the step-count feedback section on the website, both in text form and graphs. Goals are not necessarily monotonically increasing. For example, if a participant is sick, and thus records low step counts for one week, the subsequent week’s goal might be lower than the goal for the week the participant was sick. A snapshot of the individual step-count webpage can be seen in Figure 4.

**Figure 5:**
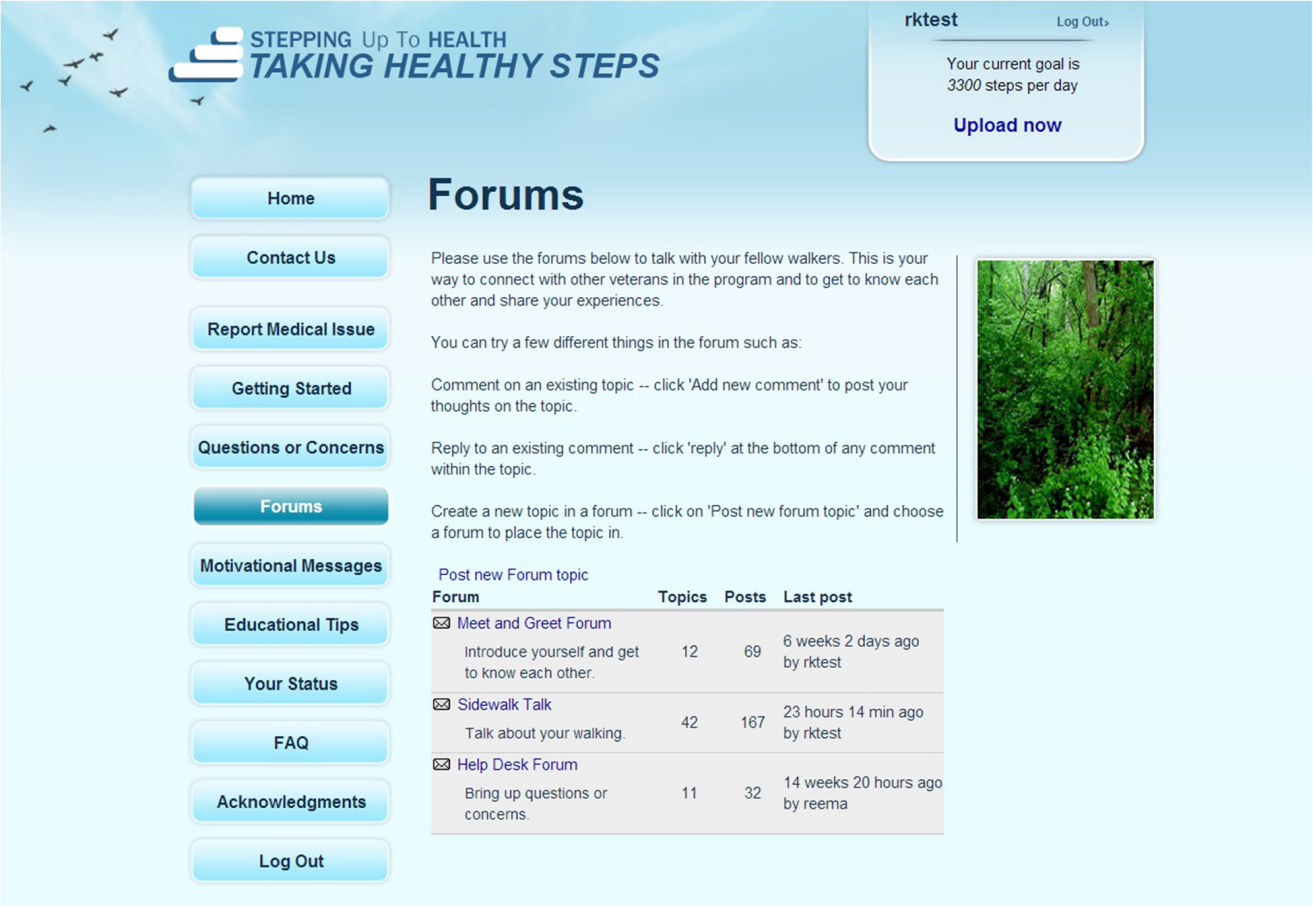
Example of the online communities and forums for participants in Taking Healthy Steps.

Additionally, all participants randomized to the intervention arm have access to *online community features*. They are able to read and post messages on the community message board under three different sections: Help Desk, Meet and Greet, and Sidewalk Talk. Study staff moderate and seed the message board. They also respond to some posts directly and stage competitions to encourage posting. The use of online communities has previously increased program retention in similar studies [47]. Finally, all participants who are randomized to the intervention arm have access to the study staff for questions, which could be initiated by sending an email or directly on the website through a form. Participants can also call the staff on a toll-free number. A screenshot of the online communities is presented in Figure 5.

### Wait list (control arm)

Wait list controls are notified that they are enrolled in the study, but that they will not be able to start the active intervention for one year. They are neither assigned step-count goals nor do they have access to the personalized dynamic website during the year long waiting period. However, they have access to a brief static webpage that provides a checklist of completed surveys and their week number in the study. Participants keep the study pedometer and are asked to log in monthly to report adverse events and to upload their pedometer data, with no other incentive to log into the website and no feedback of pedometer data or goal setting. They are given no specific instructions on exercise.

### Outcomes and data collection procedures

The *primary outcome* is change in Saint George’s Respiratory Questionnaire (SGRQ) Total Score at 4 and 12 months compared to baseline. The SGRQ is a well validated disease-specific HRQL measure [48] widely used in clinical trials of pulmonary rehabilitation, disease management interventions, and exercise [49] in patients with COPD. SGRQ will be measured at baseline, four months, and one year after randomization.

Hospitalizations for AECOPD, self-reported dyspnea, and change in average daily step counts will be *secondary outcomes*. To ascertain hospital admissions, a two-way process will be performed, using both self-reported hospitalizations (which capture non-VA admissions) and identifying hospital admissions through the VA administrative databases. ICD-9 codes will be used to identify cause of admission. AECOPD will be defined by a previously validated combination of inpatient and outpatient ICD-9 codes [50], and validated algorithms will also be used to identify pneumonias and episodes of respiratory failure [51,52]. Dyspnea is measured with the MMRC Dyspnea Score [53,54]. Change in average daily step counts will be calculated using 7 days where at least 5 of the 7 days have at least 100 steps and 8 hours of activity. Goal commitment (percentage of intervention days during which step-count goals are achieved, as well as self-reported goal commitment and perceived difficulty of goal level evaluated by the Likert scale and open-ended questions) and participant retention are also secondary outcomes. Endpoints are evaluated at baseline, four months, and one year after randomization.

### Power analysis and sample size

The sample size is calculated based on a between-arm comparison of the primary outcome – change in SGRQ total score over four months – and to allow for a comparison between rural vs. urban Veterans within the intervention group. In order to have adequate power for the comparison between rural vs. urban Veterans within intervention group, randomization is done in a 2:1 proportion to the intervention arm. We made the following assumptions, all of which are supported by the literature. First, as the minimal clinically significant mean change is SGRQ of 4 units [55], we assume at least a 4-point drop in SGRQ in the intervention arm, a conservative estimate of what might be expected with pulmonary rehabilitation or physical activity interventions in patients with COPD [56,57]. In contrast, wait list controls are expected to experience some deterioration in SGRQ even over a short period of time. In one study of 624 patients with COPD, SGRQ steadily worsened over time with an increase of approximately 2 units per year. The reported standard deviation of the change in SGRQ is approximately 10 for the intervention and wait list control groups over four-months. Thus to detect with 80% power a conservatively estimated between-arm difference in mean change of 4.3 units using a two-tailed 0.05-level test based on regression analysis and assuming a 2:1 ratio of enrollment, we estimated that 192 evaluable participants would be needed in total, with 128 randomized to the intervention arm and 64 to the control arm. The proposed sample size would provide 80% power to detect a difference within intervention arm between rural vs. urban Veterans of 5 units or larger assuming a standard deviation of 10. Assuming a 20% dropout rate, a total of about 240 participants will be recruited.

### Monitoring and reporting of adverse events

Participants can report an adverse event (AE) by phone, website, or email, and are invited to do so every 30 days. The AEs are tracked electronically in a participant management system and classified as serious if they require hospitalization or result in significant disability or classified as minor if they result in minimal or no disability and do not require hospitalization.

### Ethical and additional safety provisions

Ethical approval for this study was granted by the VA Ann Arbor Healthcare System Human Studies Sub-committee. Additionally, unsupervised home-based exercise programs in the chronically ill elderly require the design of safety provisions to maximize the positive impact, while decreasing the chances of adverse events. The current clinical trial includes preventative measures to increase safety, such as pre-inclusion evaluation and clearance by the personal physician, educational content on the website about the proper shoes and socks to wear, and weather-related recommendations. In order to prevent inappropriate or offensive interactions online, all community posts are reviewed for content within 48 hours and removed if the research staff considers the post offensive or hostile or to protect personally identifying health information. Participants can also report concerns about other participant’s posts to the study staff.

### Analysis plan

Descriptive statistics including proportions or means and their standard deviations are used, when appropriate, to describe various participant characteristics by study groups, such as demographics, symptoms, place of residency (rural vs. urban), and SGRQ scores at baseline (randomization) as well as outcome variables including changes in SGRQ total scores at 4 months and one year. The primary aim is to compare HRQL at four months and at one year in the intervention group compared to the wait list control group whose exposure to the intervention is delayed. Analyses will use the intent-to-treat approach in which the person is considered to be in his/her randomly assigned condition no matter how much he/she participates in it. Separate regression analysis will be done using changes in SGRQ from baseline to four months as the response variable and also using changes from baseline to one year as the response variable. Each regression model will include as primary predictor an indicator for the intervention group and will be adjusted for the baseline values of the response variable and the balancing factors of breathlessness and place of residence. The SGRQ is normally distributed in the COPD population and has true interval scale properties, and thus it is appropriate as a response variable in regression analyses [58]. Additionally, baseline characteristics including age, sex, COPD severity, baseline step counts, and current smoking status will be tested for any imbalances between study groups and/or for potential confounding and will be included in the regression model as appropriate to adjust for confounding.

For the secondary outcome of number of days of hospitalization during the year following randomization, we will use a Poisson regression or a negative binomial regression to make the between-group comparison, with days of hospitalization as the outcome variable, number of follow-up days as the offset variable, and an indicator for the intervention arm as the primary predictor. The model will be adjusted for breathlessness, place of residence and potential confounders, including age, sex, and COPD severity. Additional secondary analyses will examine ER visits, days of hospitalization for COPD-specific diagnoses, ICU days, and total number of admissions using the same methods.

## Results

### Baseline characteristics of participants

Mailed recruitment materials were sent to 28,957 Veterans identified from the administrative database, and 779 provided response and were screened for eligibility (response rate of 2.7%). The most common reason for ineligibility was self-reported moderate physical activity greater than 150 minutes a week. After receiving consent from 307 participants, 239 were randomized: 155 to the intervention and 84 to the wait list control. The recruitment of patients and reasons for exclusion are in the Consort diagram (Figure 2). The demographic, clinical characteristics, baseline step counts, and HRQL measurements are described in Table 1. There was no significant difference in baseline characteristics between the two arms. In general, participants are older (mean age 66.7 ± 8.8); predominantly male (93.7%); self-described as retirees (58.2%) or not currently working (24.7%); and almost half live in rural or remote areas (45.2%); and 25.1% currently smoke. The clinical characteristics include a high number of comorbidities (mean 4.9 per patient), moderate to severe dyspnea in 30.5%, and a total SGRQ score of 46.0 ± 15.4 points. The average baseline daily step counts is 3,497 ± 2,220 steps.

**Table 1:** Baseline characteristics of the participants in taking healthy steps

	**Intervention group (n = 155)**	**Control group (n = 84)**	**Total (n = 239)**	**p-value**
**Demographics**				
Age in years (mean [s.d.])	66.9 (8.7)	66.4 (9.2)	66.7 (8.8)	0.71
Male gender (n [%])	147 (94.8)	77 (91.7)	224 (93.7)	0.33
Race/Ethnicity (n [%])				0.88
*Black*	7 (4.5)	3 (3.6)	10 (4.2)	
*White*	143 (92.3)	79 (94.0)	222 (92.9)	
*Other/combined*	5 (3.3)	2 (2.4)	7 (2.9)	
Hispanic	5 (3.3)	1 (1.2)	6 (2.5)	0.34
**Socioeconomics**				
Community setting (n [%])				0.79
*Urban*	84 (54.2)	47 (56.0)	131 (54.8)	
*Rural*	71 (45.8)	37 (44.1)	108 (45.2)	
Employment (n [%])				0.79
*Retired*	94 (60.7)	45 (53.6)	139 (58.2)	
*Full-Time*	14 (9.0)	7 (8.3)	21 (8.8)	
*Part-Time*	10 (6.5)	6 (7.1)	16 (6.7)	
*Ill, disabled or unemployed*	35 (22.6)	24 (28.5)	59 (28.6)	
*Other*	2 (1.3)	2 (2.4)	4 (1.7)	
Education (n [%])				0.29
*Below high school*	4 (2.6)	2 (2.4)	6 (2.5)	
*High school or GED*	34 (22.2)	11 (13.3)	45 (19.1)	
*Vocational/Technical*	25 (16.3)	11 (13.3)	36 (15.3)	
*Some college*	52 (34.0)	29 (34.9)	81 (34.3)	
*College graduate*	38 (24.8)	30 (36.1)	68 (28.8)	
Annual income (n [%])				0.34
*Below $30 k*	66 (42.6)	35 (41.7)	101 (42.3)	
*$30 to < $40 k*	23 (14.8)	14 (16.7)	37 (15.5)	
*$40 to < $50 k*	21 (13.6)	5 (6.0)	26 (10.9)	
*> $50 k*	24 (15.5)	19 (22.6)	43 (18.0)	
*No answer*	21 (13.6)	11 (13.1)	32 (13.4)	
**Clinical characteristics**				
Ever-smokers (n [%])	146 (94.2)	77 (91.7)	223 (93.3)	0.48
*Current smokers (n [%])*	42 (27.1)	18 (21.4)	60 (25.1)	0.34
Oxygen use (n [%])	35 (22.6)	21 (25.0)	56 (23.4)	0.67
General health (n [%])				0.49
*Excellent, very good*	9 (5.8)	9 (10.7)	18 (7.5)	
*Good*	49 (31.6)	26 (31.0)	75 (31.4)	
*Fair or poor*	96 (61.9)	49 (58.3)	145 (60.7)	
*No answer*	1 (0.7)	0 (0.0)	1 (0.4)	
SGRQ score (mean [s.d.])				
*Total score*	45.5 (15.4)	46.8 (15.6)	46.0 (15.4)	0.54
*Symptoms score*	56.9 (19.4)	56.0 (19.9)	56.6 (19.5)	0.72
*Activity score*	62.1 (20.3)	64.2 (18.0)	62.8 (19.5)	0.44
*Impact score*	32.2 (16.4)	34.1 (17.9)	32.9 (16.9)	0.41
Dyspnea (MMRC score) (mean [s.d.])				0.69
*0-1*	109 (70.3)	57 (67.9)	166 (69.5)	
*2-4*	46 (29.7)	27 (32.1)	73 (30.5)	
Comorbidities (mean [s.d.])	4.8 (2.6)	4.9 (2.6)	4.9 (2.6)	0.82
Baseline step counts				
*Step counts (mean [s.d.])*	3484 (2309)	3521 (2058)	3497 (2220)	0.90

## Discussion

Patients with COPD experience progressive deterioration in their functional status, HRQL, and ability to exercise. Although continuous exposure to tobacco and poor lung function are strong factors related to poor outcomes in this population, the presence of comorbidities has emerged as a significant factor associated with all unfavorable COPD outcomes. Lack of physical activity is both a consequence and a mediator of COPD outcomes, and the success of pulmonary rehabilitation in decreasing hospital admissions, improving HRQL, and decreasing dyspnea has made rehabilitation one of the cornerstones of COPD management. Unfortunately, many barriers limit initial participation in rehabilitation, and there is a need for reinforcement to maintain the gains achieved by patients after completion of a formal program. Veterans with COPD face these barriers, which are complicated by the high frequency of rural residency, comorbidity, and unemployment in this population. Building on previous research that has shown the feasibility of using a pedometer by COPD patients, the accuracy of the device, the ability to individualize and have feedback of an automated intervention, and patients’ favorable views towards participating in online communities, we designed the current pedometer-enhanced, Internet-mediated clinical trial of physical activity for Veterans with COPD. The clinical trial includes unique characteristics such as identification of the pool of potential participants using administrative databases, remote orientation on the use of the pedometer, patient-initiated acquisition of medical clearance for participation, automated web-based data collection, use of multiple communication tools (e.g. pedometer, participation in online communities), and a dynamic, adaptive algorithm for step-count goals.

Our baseline (enrollment) data suggest that by using remote communication tools it is feasible to recruit often hard-to-reach individuals, including: rural and remote residents; elderly subjects; the unemployed; and those with multiple comorbidities [59]. Follow-up at four months and one year are required to evaluate the primary and secondary outcomes (HRQL, step counts, dyspnea, exacerbations and hospitalizations), and the retention and satisfaction with the program. Taking Healthy Steps will also be implemented after one year for wait list controls. Potential limitations of the current design include the low response to the mailed invitation, but this is no different from other studies of investigator-initiated mail contact with COPD patients [27], and the absence of spirometry for confirmation of COPD. In addition, the findings may not be generalizable to women and other patients with COPD who have Internet access but choose not to participate. Participants need some degree of familiarity with the use of computers and information technology, but as the penetration of the Internet within the chronically ill has increased [60], the potential use of the program could increase. Despite these limitations, this study will provide additional answers and insights to further develop, refine, test, and disseminate effective, low-cost behavioral interventions that improve HRQL in the older patients with multiple comorbidities who live in remote areas.

## Abbreviations

COPD: Chronic obstructive pulmonary disease; AECOPD: Acute exacerbations; HRQL: Health-related quality of life; NNT: Number-needed-to-treat; RUCA: Rural urban commuting codes; MMRC: Modified medical research council; SGRQ: Saint George’s respiratory questionnaire; AE: Adverse event.

## Competing interests

The authors declare that they have no competing interests.

## Authors’ contributions

MLM, HQN, MC, RK, DEG, NDG, CRR were involved in the conception and design of all stages of the study. CHM, MLM, HQN, MC, RK, PR, HMK, CRR were involved in study data collection. CHM, RK, PR, RGH, HMK, NDG, conducted study analyses. All authors read and approved the final manuscript.

## Pre-publication history

The pre-publication history for this paper can be accessed here:

http://www.biomedcentral.com/1471-2466/14/12/prepub

## References

[B1] Halbert RJ Natoli JL Gano A Badamgarav E Buist AS Mannino DM Global burden of COPD: systematic review and meta-analysis Eur Respir J 2006 28 523 532 10.1183/09031936.06.00124605 16611654

[B2] Buist AS McBurnie MA Vollmer WM Gillespie S Burney P Mannino DM Menezes AM Sullivan SD Lee TA Weiss KB Jensen RL Marks GB Gulsvik A Nizankowska-Mogilnicka E International variation in the prevalence of COPD (the BOLD Study): a population-based prevalence study Lancet 2007 370 741 750 10.1016/S0140-6736(07)61377-4 17765523

[B3] Centers for Disease Control Prevention Deaths from chronic obstructive pulmonary disease--United States, 2000–2005 MMWR Morb Mortal Wkly Rep 2008 57 1229 1232 19008792

[B4] Sharafkhaneh A Petersen NJ Yu HJ Dalal AA Johnson ML Hanania NA Burden of COPD in a government health care system: a retrospective observational study using data from the US Veterans Affairs population Int J Chron Obstruct Pulmon Dis 2010 5 125 132 20461144 10.2147/copd.s8047 PMC2866562

[B5] Donaldson GC Seemungal TA Bhowmik A Wedzicha JA Relationship between exacerbation frequency and lung function decline in chronic obstructive pulmonary disease Thorax 2002 57 847 852 10.1136/thorax.57.10.847 12324669 PMC1746193

[B6] Miravitlles M Ferrer M Pont A Zalacain R Alvarez-Sala JL Masa F Verea H Murio C Ros F Vidal R Effect of exacerbations on quality of life in patients with chronic obstructive pulmonary disease: a 2 year follow up study Thorax 2004 59 387 395 10.1136/thx.2003.008730 15115864 PMC1746989

[B7] Hurst JR Vestbo J Anzueto A Locantore N Mullerova H Tal-Singer R Miller B Lomas DA Agusti A Macnee W Calverley P Rennard S Wouters EF Wedzicha JA Susceptibility to exacerbation in chronic obstructive pulmonary disease N Engl J Med 2010 363 1128 1138 10.1056/NEJMoa0909883 20843247

[B8] Burge PS Calverley PM Jones PW Spencer S Anderson JA Maslen TK Randomised, double blind, placebo controlled study of fluticasone propionate in patients with moderate to severe chronic obstructive pulmonary disease: the ISOLDE trial BMJ 2000 320 1297 1303 10.1136/bmj.320.7245.1297 10807619 PMC27372

[B9] Pauwels RA Lofdahl CG Laitinen LA Schouten JP Postma DS Pride NB Ohlsson SV Long-term treatment with inhaled budesonide in persons with mild chronic obstructive pulmonary disease who continue smoking. European respiratory society study on chronic obstructive pulmonary disease N Engl J Med 1999 340 1948 1953 10.1056/NEJM199906243402503 10379018

[B10] Decramer M Celli B Kesten S Lystig T Mehra S Tashkin DP Effect of tiotropium on outcomes in patients with moderate chronic obstructive pulmonary disease (UPLIFT): a prespecified subgroup analysis of a randomised controlled trial Lancet 2009 374 1171 1178 10.1016/S0140-6736(09)61298-8 19716598

[B11] Jenkins CR Jones PW Calverley PM Celli B Anderson JA Ferguson GT Yates JC Willits LR Vestbo J Efficacy of salmeterol/fluticasone propionate by GOLD stage of chronic obstructive pulmonary disease: analysis from the randomised, placebo-controlled TORCH study Respir Res 2009 10 59 10.1186/1465-9921-10-59 19566934 PMC2714501

[B12] Jones PW Health status and the spiral of decline COPD 2009 6 59 63 10.1080/15412550802587943 19229709

[B13] Celli B Decramer M Kesten S Liu D Mehra S Tashkin DP Mortality in the 4-year trial of tiotropium (UPLIFT) in patients with chronic obstructive pulmonary disease Am J Respir Crit Care Med 2009 180 948 955 10.1164/rccm.200906-0876OC 19729663

[B14] Martinez CH Han MK Contribution of the environment and comorbidities to chronic obstructive pulmonary disease phenotypes Med Clin North Am 2012 96 713 727 10.1016/j.mcna.2012.02.007 22793940 PMC4629222

[B15] Effing TW Bourbeau J Vercoulen J Apter AJ Coultas D Meek P Valk P Partridge MR Palen J Self-management programmes for COPD: moving forward Chron Respir Dis 2012 9 27 35 10.1177/1479972311433574 22308551

[B16] Watz H Waschki B Meyer T Magnussen H Physical activity in patients with COPD Eur Respir J 2009 33 262 272 19010994 10.1183/09031936.00024608

[B17] Pitta F Breyer MK Hernandes NA Teixeira D Sant’Anna TJ Fontana AD Probst VS Brunetto AF Spruit MA Wouters EF Burghuber OC Hartl S Comparison of daily physical activity between COPD patients from Central Europe and South America Respir Med 2009 103 421 426 10.1016/j.rmed.2008.09.019 19006659

[B18] Lehouck A Boonen S Decramer M Janssens W COPD, bone metabolism, and osteoporosis Chest 2011 139 648 657 10.1378/chest.10-1427 21362651

[B19] Hopkinson NS Tennant RC Dayer MJ Swallow EB Hansel TT Moxham J Polkey MI A prospective study of decline in fat free mass and skeletal muscle strength in chronic obstructive pulmonary disease Respir Res 2007 8 25 10.1186/1465-9921-8-25 17355636 PMC1832189

[B20] Schnell K Weiss CO Lee T Krishnan JA Leff B Wolff JL Boyd C The prevalence of clinically-relevant comorbid conditions in patients with physician-diagnosed COPD: a cross-sectional study using data from NHANES 1999–2008 BMC Pulm Med 2012 12 26 10.1186/1471-2466-12-26 22695054 PMC3461433

[B21] Moy ML Teylan M Weston NA Gagnon DR Garshick E Daily step count predicts acute exacerbations in a US cohort with COPD PLoS One 2013 8 e60400 10.1371/journal.pone.0060400 23593211 PMC3617234

[B22] Waschki B Kirsten A Holz O Muller KC Meyer T Watz H Magnussen H Physical activity is the strongest predictor of all-cause mortality in patients with COPD: a prospective cohort study Chest 2011 140 331 342 10.1378/chest.10-2521 21273294

[B23] Moy ML Teylan M Weston NA Gagnon DR Danilack VA Garshick E Daily step count is associated with plasma CRP and IL-6 in a US cohort with COPD Chest 2013 doi: 10.1378/chest.13-1052. [Epub ahead of print] 10.1378/chest.13-1052 24091482

[B24] Ries AL Bauldoff GS Carlin BW Casaburi R Emery CF Mahler DA Make B Rochester CL Zuwallack R Herrerias C Pulmonary rehabilitation: joint ACCP/AACVPR evidence-based clinical practice guidelines Chest 2007 131 Suppl 5 S4 42 10.1378/chest.06-2418 17494825

[B25] Puhan MA Gimeno-Santos E Scharplatz M Troosters T Walters EH Steurer J Pulmonary rehabilitation following exacerbations of chronic obstructive pulmonary disease Cochrane Database Syst Rev 2011 10 CD005305 doi: 10.1002/14651858.CD005305.pub3. Review 21975749 10.1002/14651858.CD005305.pub3

[B26] Ries AL Kaplan RM Myers R Prewitt LM Maintenance after pulmonary rehabilitation in chronic lung disease: a randomized trial Am J Respir Crit Care Med 2003 167 880 888 10.1164/rccm.200204-318OC 12505859

[B27] Martinez CH Raparla S Plauschinat CA Giardino ND Rogers B Beresford J Bentkover JD Schachtner-Appel A Curtis JL Martinez FJ Han MK Gender differences in symptoms and care delivery for chronic obstructive pulmonary disease J Womens Health (Larchmt) 2012 21 1267 1274 10.1089/jwh.2012.3650 23210491 PMC3518541

[B28] Fischer MJ Scharloo M Abbink JJ Thijs-Van A Rudolphus A Snoei L Weinman JA Kaptein AA Participation and drop-out in pulmonary rehabilitation: a qualitative analysis of the patient’s perspective Clin Rehabil 2007 21 212 221 10.1177/0269215506070783 17329278

[B29] Abrams TE Vaughan-Sarrazin M Fan VS Kaboli PJ Geographic isolation and the risk for chronic obstructive pulmonary disease-related mortality: a cohort study Ann Intern Med 2011 155 80 86 10.7326/0003-4819-155-2-201107190-00003 21768581

[B30] Goodridge D Lawson J Rennie D Marciniuk D Rural/urban differences in health care utilization and place of death for persons with respiratory illness in the last year of life Rural Remote Health 2010 10 1349 20438281

[B31] Thorpe O Johnston K Kumar S Barriers and enablers to physical activity participation in patients with COPD: a systematic review J Cardiopulm Rehabil Prev 2012 32 359 369 10.1097/HCR.0b013e318262d7df 22941449

[B32] Johnston K Grimmer-Somers K Pulmonary rehabilitation: overwhelming evidence but lost in translation? Physiother Can 2010 62 368 373 10.3138/physio.62.4.368 21886377 PMC2958065

[B33] Nguyen HQ Carrieri-Kohlman V Rankin SH Slaughter R Stulbarg MS Is Internet-based support for dyspnea self-management in patients with chronic obstructive pulmonary disease possible? Results of a pilot study Heart Lung 2005 34 51 62 10.1016/j.hrtlng.2004.06.005 15647734

[B34] Borycki E M-health: can chronic obstructive pulmonary disease patients use mobile phones and associated software to self-manage their disease? Stud Health Technol Inform 2012 172 79 84 22910504

[B35] Moy ML Janney AW Nguyen HQ Matthess KR Cohen M Garshick E Richardson CR Use of pedometer and internet-mediated walking program in patients with chronic obstructive pulmonary disease J Rehabil Res Dev 2010 47 485 496 10.1682/JRRD.2009.07.0091 20803392 PMC3018275

[B36] Moy ML Weston NA Wilson EJ Hess ML Richardson CR A pilot study of an internet walking program and pedometer in COPD Respir Med 2012 106 1342 1350 10.1016/j.rmed.2012.06.013 22795984

[B37] Cameron L, Leventhal H The self-regulation of health and illness behavior 2003 London: Routledge

[B38] Boekaerts M, Pintrich P, Zeidner M Handbook of self-regulation 2000 San Diego: Academic Press

[B39] Locke E Latham G A theory of goalsetting and task performance 1990 Englewoods Cliff: Prentice-Hall

[B40] Richardson CR Mehari KS McIntyre LG Janney AW Fortlage LA Sen A Strecher VJ Piette JD A randomized trial comparing structured and lifestyle goals in an internet-mediated walking program for people with type 2 diabetes Int J Behav Nutr Phys Act 2007 4 59 10.1186/1479-5868-4-59 18021411 PMC2212636

[B41] Cooke CR Joo MJ Anderson SM Lee TA Udris EM Johnson E Au DH The validity of using ICD-9 codes and pharmacy records to identify patients with chronic obstructive pulmonary disease BMC Health Serv Res 2011 11 37 10.1186/1472-6963-11-37 21324188 PMC3050695

[B42] Hart LG Larson EH Lishner DM Rural definitions for health policy and research Am J Public Health 2005 95 1149 1155 10.2105/AJPH.2004.042432 15983270 PMC1449333

[B43] Krein SL Metreger T Kadri R Hughes M Kerr EA Piette JD Kim HM Richardson CR Veterans walk to beat back pain: study rationale, design and protocol of a randomized trial of a pedometer-based internet mediated intervention for patients with chronic low back pain BMC Musculoskelet Disord 2010 11 205 10.1186/1471-2474-11-205 20836856 PMC2945952

[B44] Holbrook EA Barreira TV Kang M Validity and reliability of Omron pedometers for prescribed and self-paced walking Med Sci Sports Exerc 2009 41 670 674 10.1249/MSS.0b013e3181886095 19204582

[B45] Danilack VA Weston NA Richardson CR Mori DL Moy ML Reasons persons with COPD do not walk and relationship with daily step count COPD 2013 [Epub ahead of print] 10.3109/15412555.2013.841670 24152213

[B46] Richardson CR Brown BB Foley S Dial KS Lowery JC Feasibility of adding enhanced pedometer feedback to nutritional counseling for weight loss J Med Internet Res 2005 7 e56 10.2196/jmir.7.5.e56 16403720 PMC1550681

[B47] Resnick PJ Janney AW Buis LR Richardson CR Adding an online community to an internet-mediated walking program. Part 2: strategies for encouraging community participation J Med Internet Res 2010 12 e72 10.2196/jmir.1339 21169161 PMC3056535

[B48] Jones PW Quirk FH Baveystock CM Littlejohns P A self-complete measure of health status for chronic airflow limitation. The St. George’s respiratory questionnaire Am Rev Respir Dis 1992 145 1321 1327 10.1164/ajrccm/145.6.1321 1595997

[B49] Paz-Diaz H Montes de Oca M Lopez JM Celli BR Pulmonary rehabilitation improves depression, anxiety, dyspnea and health status in patients with COPD Am J Phys Med Rehabil 2007 86 30 36 10.1097/PHM.0b013e31802b8eca 17304686

[B50] Mortensen EM Copeland LA Pugh MJ Restrepo MI de Molina RM Nakashima B Anzueto A Impact of statins and ACE inhibitors on mortality after COPD exacerbations Respir Res 2009 10 45 10.1186/1465-9921-10-45 19493329 PMC2697974

[B51] Joo MJ Au DH Fitzgibbon ML Lee TA Inhaled corticosteroids and risk of pneumonia in newly diagnosed COPD Respir Med 2010 104 246 252 10.1016/j.rmed.2009.10.002 19879745

[B52] Cannon KT Sarrazin MV Rosenthal GE Curtis AE Thomas KW Kaldjian LC Use of mechanical and noninvasive ventilation in black and white chronic obstructive pulmonary disease patients within the veterans administration health care system Med Care 2009 47 129 133 10.1097/MLR.0b013e3181809150 19106742

[B53] Mahler DA Ward J Waterman LA Baird JC Longitudinal changes in patient-reported dyspnea in patients with COPD COPD 2012 9 522 527 10.3109/15412555.2012.701678 22876883

[B54] Mahler DA Waterman LA Ward J McCusker C ZuWallack R Baird JC Validity and responsiveness of the self-administered computerized versions of the baseline and transition dyspnea indexes Chest 2007 132 1283 1290 10.1378/chest.07-0703 17646223

[B55] Jones PW Interpreting thresholds for a clinically significant change in health status in asthma and COPD Eur Respir J 2002 19 398 404 10.1183/09031936.02.00063702 11936514

[B56] Finnerty JP Keeping I Bullough I Jones J The effectiveness of outpatient pulmonary rehabilitation in chronic lung disease: a randomized controlled trial Chest 2001 119 1705 1710 10.1378/chest.119.6.1705 11399694

[B57] Elci A Borekci S Ovayolu N Elbek O The efficacy and applicability of a pulmonary rehabilitation programme for patients with COPD in a secondary-care community hospital Respirology 2008 13 703 707 10.1111/j.1440-1843.2008.01327.x 18713091

[B58] Jones P Lareau S Mahler DA Measuring the effects of COPD on the patient Respir Med 2005 99 Suppl B S11 18 16236492 10.1016/j.rmed.2005.09.011

[B59] Zulman DM Sussman JB Chen X Cigolle CT Blaum CS Hayward RA Examining the evidence: a systematic review of the inclusion and analysis of older adults in randomized controlled trials J Gen Intern Med 2011 26 783 790 10.1007/s11606-010-1629-x 21286840 PMC3138606

[B60] Fox S Purcell K Chronic disease and the internet [http://www.pewinternet.org/Reports/2010/Chronic-Disease.aspx]. Accessed date, 7/17/13

